# Man-made reactive oxygen species as green disinfectants

**DOI:** 10.1016/j.eehl.2023.05.001

**Published:** 2023-05-10

**Authors:** Chengjun Li, Huan Zhong, Guorui Liu, Di Liu, Mengjie Wu, Su Shiung Lam, Christian Sonne

**Affiliations:** a Institute of Environmental Research at Greater Bay Area, Guangzhou University, Guangzhou 510006, China; b School of Environment, Nanjing University, Nanjing 210023, China; c Environmental and Life Sciences Program (EnLS), Trent University, Peterborough, Ontario, Canada; d State Key Laboratory of Environmental Chemistry and Ecotoxicology, Research Center for Eco-Environmental Sciences, Chinese Academy of Sciences, Beijing 100085, China; e CAS Key Laboratory of Special Pathogens and Biosafety, Wuhan Institute of Virology, Center for Biosafety Mega-Science, Chinese Academy of Sciences, Wuhan 430071, China; f Higher Institution Centre of Excellence (HICoE), Institute of Tropical Aquaculture and Fisheries (AKUATROP), Universiti Malaysia Terengganu, 21030 Kuala Nerus, Terengganu, Malaysia; g University Centre for Research and Development, Department of Chemistry, Chandigarh University, Gharuan, Mohali, Punjab, India; h Aarhus University, Roskilde, Denmark

## Abstract

• The massive applications of disinfectants threat wildlife and humans.
• Man-made reactive oxygen species (ROS) are green alternatives for chemical disinfectants.
• *In vitro* ROS kill pathogens yet produce little to no residue.

## Graphical abstract

**Figure:**
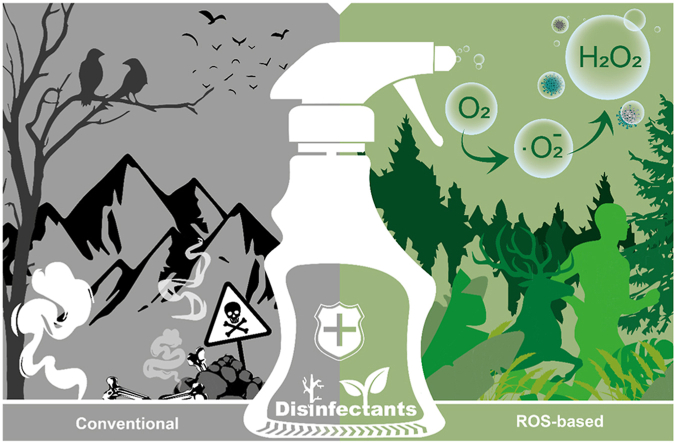

The ongoing pandemics boost the demand for chemical disinfectants, including surface disinfectants and hand sanitizers [1]. This is largely driven by increasing public health awareness and hygiene standards in public and private settings [2]. The global surface disinfectant market size in 2019 was valued at US$3.4 billion and estimated to experience a 6.0% compound annual growth rate, reaching US$5.42 billion in 2027 [3]. However, according to an updated report released in 2021, the global market size for surface disinfectants was already valued at US$5.1 billion and projected to reach US$7.36 billion by 2027. This trend is even more obvious in the hand sanitizer market (Fig. 1) [4,5]. In the U.S. alone, the hand sanitizer market experienced a dramatic 72.5% increase in 2020 compared to that in 2019, reaching US$1.38 billion [6]. Such differences between estimations made before and during the COVID-19 pandemic suggest a soaring demand for disinfectants that could linger for a long time.

**Fig. 1:**
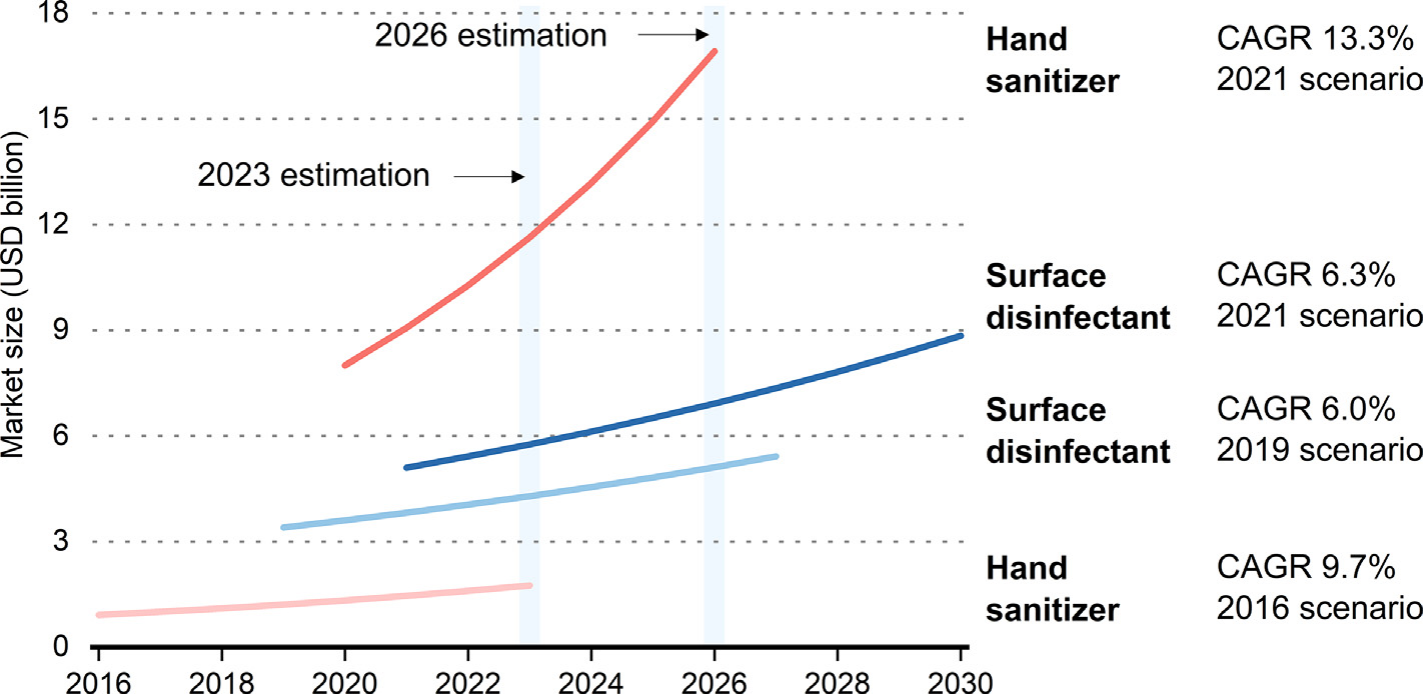
The global disinfectant market and its size (including hand sanitizer and surface disinfectant) estimated in four different scenarios as described in refs [[3], [4], [5], [6]] before and during the COVID-19 pandemic. In 2019, the global surface disinfectant market size was valued at US$3.4 billion and estimated to experience a 6.0% compound annual growth rate (CAGR) from 2020 to 2027, reaching US$3.82 billion and US$5.42 billion in 2021 and 2027, respectively [3]. Due to the surging demand, the global market size for surface disinfectants was already valued at US$5.1 billion in 2021 and projected to reach US$7.36 billion by 2027, growing at a CAGR of 6.3% from 2022 to 2030. As for the global hand sanitizer market, it was valued at US$0.919 billion in 2016 and anticipated to grow to US$1.755 billion by 2023 [4]. An updated estimation in 2021, however, suggested that the global market for hand sanitizers already surpassed US$8 billion in 2020 and is expected to fall at US$17 billion by 2026 [5].

The surging demands and massive applications of disinfectants pose severe threats to wildlife and humans during the pandemic and beyond [7,8]. The effective ingredients of common disinfectants include quaternary ammonium compounds (QACs), hydrogen peroxide, sodium hypochlorite, and alcohol. Panic and aggressive applications of disinfectants during the pandemic have released enormous amounts of these chemicals into the environment, polluting the air, water bodies, and soils [9,10]. The level of indoor QACs, for example, was almost doubled in the air compared to the pre-pandemic era [9], potentially triggering asthma, inflammation, skin irritation, decreased fertility, and so on [1,9,10]. Residues of these chemicals could also form hazardous by-products [10], killing soil and aquatic organisms [[10], [11], [12]]. Many of these by-products have been shown to alter microbial communities and persistently promote bacterial evolution toward antimicrobial resistance, leading to long-term impacts on the biota and ultimately threatening human health [12].

This is particularly worrisome in urban areas where indiscriminate and extensive use of chemical disinfectants became the norm during the pandemic. Such routine disinfection schemes threaten pets and other urban wildlife upon the bioaccumulation of residues and by-products in food webs [7]. While the acute adverse impacts of excessive use of chemical disinfectants have been widely reported, evaluations of their long-term effects are scarce and challenging. Such a dilemma is partly due to the lack of minoring data on disinfectant residues and by-products in environmental media, where lives are continuously exposed to those chemicals [10]. These call for next-generation disinfection methods that are both effective and environmentally friendly [8].

Man-made reactive oxygen species (ROS) are promising and eco-friendly alternatives for conventional chemical disinfectants. ROS generated in the air hamper the transmission of flu during summer time [13,14]. For instance, the half-lives for the virus particles in a typical droplet could be dramatically reduced to less than 1 s in the air [14]. Man-made ROS are also shown to help eliminate pathogenic bacteria and even viruses in wastewater [[15], [16], [17]]. This is because ROS attack can lead to peroxidation of the lipid membrane, depresses the activity of some periplasmic enzyme, and directly interact and damage their genetic materials by protein denaturation and amino acid oxidation [13]. This novel disinfection method produces little to no residue as environmental ROS are typically quenched by ubiquitous water molecules within milliseconds, outmatching conventional chemical disinfectants in environmental impacts [18]. Compared to vaccination, ROS-mediated virus killing could be less sensitive to variants, including those with increased immune escape properties [13]. Another major advantage of utilizing ROS as novel disinfectants is their non-selective, broad-spectrum pathogen killing, which would be of great importance in the case scenario of simultaneous multi-pathogen emergence [19].

Now, the possibility of utilizing man-made ROS as novel measures to control pathogen transmission is further increased by the advances in cutting-edge *in vitro* technologies, e.g., cold atmospheric plasma and ROS-generating materials [20]. In cold atmospheric plasma, the generation of a wide variety of ROS can be fine-tuned through the optimization of parameters, including feeding gas, energy source, and so on. [20] Similarly, controlled release of ROS can be achieved by novel ROS-generating materials [[21], [22], [23]]. These serve as therapeutic mediators generating ROS at target sites to reduce pathogen loads as well as coatings of food wrapping, medical utensils, and air-purifying biofilters, and so on for transmission prevention [[20], [21], [22]].

Such applications are promising to be implemented in closed settings, including airports, hotels, and hospitals where airflow is limited and strict hygiene and disinfection are required. Man-made ROS also have the promise for disinfection on farms that are potential incubators of many diseases. This is exemplified by the fresh pandemic fears triggered by the bird flu outbreak in captive mink [24]. Incorporating ROS-generating facilities into disinfection schemes in such settings would provide a greener and more effective solution to the emergence of variants with high spillover risks, without concerns of detrimental impacts of residues and by-products on the biota and human health.

ROS are evolutionary barriers against pathogenic infections *in vivo*, and now we are seizing the opportunity to take full advantage of such natural powers to mitigate pathogen transmission *in vitro*. Pathogen killing or virulence attenuation by man-made ROS has the potential to be a groundbreaking and environment-friendly disinfection solution. Policymakers and research scientists should work together on the swift development and application of man-made ROS and similar *in vitro* techniques as novel and sustainable measures to better control infectious diseases. These *in vitro* measures, together with vaccination campaigns, are envisaged to help fight emerging and future public health events. The efforts will be of substantial importance for the global community to achieve the Sustainable Development Goals by 2030, and ultimately pave our way toward One Health [25,26].

## Author contributions

C.L., H.Z., and C.S. identified the topic of this paper. C.L., G.L., and D.L. collected and analyzed data. C.L. and M.W. interpreted the results and designed the figures. C.L., H.Z., C.S., and S.S.L. led the manuscript writing and revision, and all the co-authors participated in the manuscript writing and/or editing. H.Z., and C.S. supervised this work.

## Declaration of competing interests

The authors declare no conflicts of interest.

## References

[1] Dewey H.M., Jones J.M., Keating M.R., Budhathoki-Uprety J. (2021). Increased use of disinfectants during the COVID-19 pandemic and its potential impacts on health and safety. ACS Chem. Health Saf..

[2] Guo J., Liao M., He B., Liu J., Hu X., Yan D., Wang J. (2021). Impact of the COVID-19 pandemic on household disinfectant consumption behaviors and related environmental concerns: a questionnaire-based survey in China. J. Environ. Chem. Eng..

[3] Million Insights (2021). https://www.millioninsights.com/industry-reports/global-surface-disinfectant-market.

[4] Research A.M. (2018). Hand sanitizer market by product (gel, foam, spray, and others), distribution channel (online store, departmental store, pharmacy store, and others), and end use (restaurants, schools, hospitals, household purpose, and others) – global opportunity Analysis and industry forecast, 2017-2023. https://www.alliedmarketresearch.com/hand-sanitizer-market.

[5] Facts and Factors (2021). Global hand sanitizer market projected to reach over USD 17 billion by 2026. https://www.fnfresearch.com/news/global-hand-sanitizer-market.

[6] Fortune Business Insights (2021). The global hand sanitizer market is projected to grow from $2.79 billion in 2021 to $3.47 billion in 2028 at a CAGR of 3.1% in forecast period, 2021-2028. https://www.fortunebusinessinsights.com/hand-sanitizer-market-102710.

[7] Nabi G., Wang Y., Hao Y., Khan S., Wu Y., Li D. (2020). Massive use of disinfectants against COVID-19 poses potential risks to urban wildlife. Environ. Res..

[8] Dhama K., Patel S.K., Kumar R., Masand R., Rana J., Yatoo M.I., Tiwari R., Sharun K. (2021). The role of disinfectants and sanitizers during COVID-19 pandemic: advantages and deleterious effects on humans and the environment. Environ. Sci. Pollut. R..

[9] Zheng G., Filippelli G.M., Salamova A. (2020). Increased indoor exposure to commonly used disinfectants during the COVID-19 pandemic. Environ. Sci. Technol. Lett..

[10] Hora P.I., Pati S.G., McNamara P.J., Arnold W.A. (2020). Increased use of quaternary ammonium compounds during the SARS-CoV-2 pandemic and beyond: consideration of environmental implications. Environ. Sci. Technol. Lett..

[11] Chu W., Fang C., Deng Y., Xu Z. (2020). Intensified disinfection amid COVID-19 pandemic poses potential risks to water quality and safety. Environ. Sci. Technol..

[12] Mantilla-Calderon D., Plewa M.J., Michoud G., Fodelianakis S., Daffonchio D., Hong P.Y. (2019). Water disinfection byproducts increase natural transformation rates of environmental DNA in Acinetobacter baylyi ADP1. Environ. Sci. Technol..

[13] Wainwright M., Maisch T., Nonell S., Plaetzer K., Almeida A., Tegos G.P., Hamblin M.R. (2017). Photoantimicrobials—are we afraid of the light?. Lancet Infect. Dis..

[14] Spooner R., Yilmaz Ö. (2011). The role of reactive-oxygen-species in microbial persistence and inflammation. Int. J. Mol. Sci..

[15] Deng Y., Zhao R. (2015). Advanced oxidation processes (AOPs) in wastewater treatment. Curr. Pollut. Rep..

[16] Nasir A.M., Awang N., Hubadillah S.K., Jaafar J., Othman M.H.D., Wan Salleh W.N., Ismail A.F. (2021). A review on the potential of photocatalysis in combatting SARS-CoV-2 in wastewater. J. Water Process Eng..

[17] Ma J., Wei Z., Spinney R., Dionysiou D.D., Xiao R. (2021). Emerging investigator series: could the superoxide radical be implemented in decontamination processes?. Environ. Sci. Water Res. Technol..

[18] Richards T., Harrhy J.H., Lewis R.J., Howe A.G.R., Suldecki G.M., Folli A., Morgan D.J., Davies T.E. (2021). A residue-free approach to water disinfection using catalytic in situ generation of reactive oxygen species. Nat. Catal..

[19] Li C., Zhong H., Xie Y., Bai T., Yan B., Sonne C. (2023). Speed up multi-pathogen surveillance. Lancet.

[20] Kaushik N., Mitra S., Baek E.J., Nguyen L.N., Bhartiya P., Kim J.H., Choi E.H., Kaushik N.K. (2022). The inactivation and destruction of viruses by reactive oxygen species generated through physical and cold atmospheric plasma techniques: current status and perspectives. J. Adv. Res..

[21] Hu L., Hou A., Xie K., Gao A. (2019). Light-induced production of reactive oxygen species by a novel water-soluble benzophenone derivative containing quaternary ammonium groups and its assembly on the protein fiber surface. ACS Appl. Mater. Interfaces.

[22] Wang Y., Xu Y., Dong S., Wang P., Chen W., Lu Z., Ye D., Pan B. (2021). Ultrasonic activation of inert poly (tetrafluoroethylene) enables piezocatalytic generation of reactive oxygen species. Nat. Commun..

[23] Ni Z., Zhang C., Ma H., Liu J., Wang Z., Zhu K., Li M., Jia H. (2022). Facet-dependent photo-degradation of nitro polycyclic aromatic hydrocarbons on hematite under visible light: Participation of environmentally persistent free radicals and reactive oxygen/nitrogen species. Appl. Catal. B Environ..

[24] Sidik S.M. (2023). Bird flu outbreak in mink sparks concern about spread in people. Nature 614.

[25] Li C., Jiang G., Ren H. (2022). The common vision toward one health. Eco-Environ. Health.

[26] Xu H., Jia Y., Sun Z., Su J., Liu Q., Zhou Q., Jiang G. (2022). Environmental pollution, a hidden culprit for health issues. Eco-Environ. Health.

